# Electrochemotherapy in the Treatment of Cutaneous Metastases from Breast Cancer: A Multicenter Cohort Analysis

**DOI:** 10.1245/s10434-015-4779-6

**Published:** 2015-08-05

**Authors:** C. Cabula, L. G. Campana, G. Grilz, S. Galuppo, R. Bussone, L. De Meo, A. Bonadies, P. Curatolo, M. De Laurentiis, M. Renne, S. Valpione, T. Fabrizio, N. Solari, M. Guida, A. Santoriello, M. D’Aiuto, R. Agresti

**Affiliations:** Oncologic Surgery, Ospedale Oncologico A. Businco, Cagliari, Italy; Veneto Institute of Oncology IOV-IRCCS, Padua, Italy; Breast Surgery Unit, Ospedale Le Molinette, Turin, Italy; Humanitas-Centro Catanese di Oncologia, Catania, Italy; Plastic Surgery Unit, San Gallicano Dermatologic Institute, Rome, Italy; Dermatology and Plastic Surgery Department, La Sapienza University, Rome, Italy; Seconda Università di Napoli, Naples, Italy; Fondazione T. Campanella, Catanzaro, Italy; Plastic Surgery Unit, IRCCS, Referral Cancer Center of Basilicata, Rionero in Vulture, Italy; Surgical Unit 1, IRCCS San Martino-IST, Genoa, Italy; Medical Oncology Unit, Istituto dei Tumori, Bari, Italy; Department of Medicine and Surgery, “Federico II” University, Naples, Italy; Breast Surgery Unit, Istituto Nazionale Tumori “Pascale”, Naples, Italy; Breast Surgery Unit, Fondazione IRCCS Istituto Nazionale dei Tumori, Milan, Italy

## Abstract

**Background:**

The management of breast cancer (BC) skin metastases represents a therapeutic challenge. Electrochemotherapy (ECT) combines the administration of bleomycin with temporary permeabilization induced by locally administered electric pulses. Preliminary experience with ECT in BC patients is encouraging.

**Methods:**

A total of 125 patients with BC skin metastases who underwent ECT between 2010 and 2013 were enrolled onto a multicenter retrospective cohort study. The treatment was administered following the European Standard Operative Procedures of Electrochemotherapy. Tumor response was clinically assessed adapting the Response Evaluation Criteria in Solid Tumors, and toxicity was evaluated according to Common Terminology Criteria for Adverse Events 4.0. Cox regression analysis was used to identify predictive factors.

**Results:**

Response was evaluable in 113 patients for 214 tumors (median 1 per patient, range 1–3). The overall response rate after 2 months was 90.2 %, while the complete response (CR) rate was 58.4 %. In multivariate analysis, small tumor size (*P* < 0.001), absence of visceral metastases (*P* = 0.001), estrogen receptor positivity (*P* = 0.016), and low Ki-67 index (*P* = 0.024) were significantly associated with CR. In the first 48 h, 10.4 % of patients reported severe skin pain. Dermatologic toxicity included grade 3 skin ulceration (8.0 %) and grade 2 skin hyperpigmentation (8.8 %). Tumor 1-year local progression-free survival was 86.2 % (95 % confidence interval 79.3–93.8) and 96.4 % (95 % confidence interval 91.6–100) in the subgroup of those with CR.

**Conclusions:**

In this study, small tumor size, absence of visceral metastases, estrogen receptor positivity, and low Ki-67 index were predictors of CR after ECT. Patients who experienced CR had durable local control. ECT represents a valuable skin-directed therapy for selected patients with BC.

Skin metastases from breast cancer (BC) are often symptomatic for ulceration, bleeding, and pain, and they may represent a challenge for clinicians, particularly in heavily pretreated patients. Surgical resection, radiotherapy, and systemic therapies can be variously combined according to individual patient characteristics, tumor features, and physician choice.^1^ When surgical excision is not possible, radiotherapy ensures sustained local control, even if this is not feasible in preirradiated areas.^2^ Systemic therapies, such as endocrine treatment, chemotherapy, and targeted agents, represent valuable options, depending on the molecular subtype of BC and prior therapies.^3^ Application of topic chemotherapy and laser ablation is limited to cancers confined to the top layer of skin.^4^ Electrochemotherapy (ECT) combines the administration of a poorly permeant cytotoxic agent, such as bleomycin (BLM), with the local application of electric pulses that induce reversible electroporation, thus improving drug diffusion into cells.^5^ ECT was introduced in 2006, demonstrating a high rate of efficacy and favorable toxicity profile in a European multicenter study on skin metastases from different tumor histotypes.^6^ In this study, the objective response (OR) rate on treated tumor nodules was 89.0 % with complete regression in 73.3 % of cases. A recently published meta-analysis including 47 prospective studies comparing five skin-directed therapies (ECT, radiation, photodynamic therapy, intralesional therapy, and topical therapy), ECT demonstrated an OR rate of 75.4 % (CR rate, 47.5 %) with a low toxicity profile (grade 3 in less than 6 % of patients).^7^ In this analysis, melanoma and BC comprised 96.8 % of all cutaneous metastases, with similar response rates.

To our knowledge, published data on ECT in BC patients with cutaneous skin metastases are based on small, single-center, heterogeneous series. Consequently, these series do not allow for identification of clinical and/or biologic factors that are reliably predictive of ECT response.^8^ The aim of our study was to provide a systemic analysis on a large series of BC patients treated with ECT, evaluating potential predictive factors of response to treatment.

## Patients and Methods

### Patients

Between January 2010 and June 2013, the Italian Senological Group for Electrochemotherapy (GISEL), involving 13 Italian institutions, performed this multicenter retrospective cohort study. Inclusion criteria for ECT included BC patients with cutaneous and/or subcutaneous histologically confirmed metastases. Exclusion criteria for ECT included tumors in close proximity to a cardiac pacemaker; allergy to BLM; prior cumulative dose of BLM exceeding 250,000 IU/m^2^; serum creatinine >150 μmol/L; lung fibrosis; and pregnancy or lactation. Patients were enrolled regardless of the presence of other metastases. The respective institutional review boards of the participating institutions approved the study. All patients gave informed consent for the procedure and for utilization of their data for scientific purposes. Clinical records were anonymously entered into a dedicated encrypted online database.

### Evaluation of Estrogen Receptor (ER), Progesterone Receptor (PgR), *HER2* Status, and Ki-67 Index

Histologic diagnosis, immunohistochemical analysis, and fluorescence in situ hybridization for *HER2* gene amplification (in case of inconclusive results on *HER2* status) were performed according to international guidelines. The cutoff for ER and PgR positivity was 1 % of cells with positive nuclear staining.^9^ Positivity for *HER2* was determined by either immunohistochemistry 3+ or fluorescence in situ hybridization amplification. The cutoff point for the Ki-67 labeling index was 14 %.^10^ Surrogate subtypes were defined according to the criteria established by the St. Gallen International Breast Cancer Conference.^11^

### Treatment

The European Standard Operative Procedures of Electrochemotherapy (ESOPE) were used for all patients.^12^ Accordingly, the dose and route of BLM administration were adapted to the number and size of tumors in case of intratumoral injection, and to the patient’s body surface area in case of intravenous infusion. The procedure was scheduled in a day-hospital regimen, and patients were usually discharged after an observation period of 24 h.

### Patient Assessment

Patients were evaluated after 1 and 2 weeks for acute toxicity and at 4 and 8 weeks for late toxicity and tumor response; subsequent follow-up visits were planned every 3–4 months. Among 125 patients, 12 (9.6 %) were followed for less than 2 months after ECT and were not considered for assessment of response.

For each patient, up to a maximum of five measurable tumors were registered as target lesions. The sum of their maximum diameters represented the baseline measurement for assessment of tumor response, which was clinically performed by Response Evaluation Criteria in Solid Tumors 1.1.^13^ In case of the presence of many confluent nodules, when it was impossible to count their exact number, they were considered as a single entity and measured as a single area of treatment. Treatment toxicity and adverse events were graded according to the Common Terminology Criteria for Adverse Events 4.^14^ Pain was graded according to a 0–10 numeric pain intensity scale (0 = no pain, 10 = maximum pain).^15^

### Statistical Analysis

In descriptive analyses, continuous variables are reported as median value and interquartile range and categorical variables are reported as absolute number and percentage.

Evaluation of tumor response was performed by contingency tables and Pearson’s *χ*^2^ test.

Survival curves were estimated by the Kaplan–Meier method and compared to the log rank test. Hazard ratios were calculated by a Cox proportional risk model, after proportional hazard assumption confirmation with Schoenfeld residuals. Local progression-free survival (LPFS) was calculated from achievement of response in the treated area to local progression of disease, including the appearance of new nodules in the same area, or last follow-up. Statistical analyses were performed by SPSS 22.0 (IBM) software.

## Results

### Patient and Disease Characteristics

Baseline patient and disease characteristics are reported in Table 1. The prevalent tumor histotype was infiltrating ductal carcinoma (76.6 %). Tumor, node, metastasis classification of primary BC was T1–T2 in 48 % patients and T3–T4 in 52 %; 67.2 % patients had lymph node involvement, and 22.4 % had distant metastases.

**Table 1:** Patient characteristics (*n* = 125)

Characteristic	Median (range) or *n* (%)
Age (years)	63 (54–72)
Histology	
IDC	97 (76.6)
Non-IDC	28 (23.4)
Time since occurrence of skin metastases (mo)	32 (9–109)
Skin metastases (*n* = 239)	
No. per patient	1 (1–3)
Size (mm)	21 (15–45)
Location	
Chest	222 (92.9)
Other site	17 (7.1)
Skin condition	
Previous radiotherapy	92 (38.5)
Lymphedema	30 (12.6)
Ulceration	64 (26.8)
Immunohistochemistry	
ER positive	72 (57.6)
PgR positive	72 (57.6)
*HER2* overexpression	35 (28.0)
Ki-67 < 14 %	63 (50.4)
Surrogate subtypes^a^	
Luminal A-like	23 (18.4)
Luminal B-like (*HER2* negative)	22 (17.6)
Luminal B-like (*HER2* positive)	18 (14.4)
Triple negative	35 (28)
*HER2*	11 (8.8)
Previous treatments^b^	
Radiotherapy	68 (54.4)
Chemotherapy	92 (73.6)
Endocrine therapy	71 (56.8)
Targeted therapy	14 (11.2)
Surgery for skin metastases	89 (71.2)

*IDC* invasive ductal carcinoma, *ER* estrogen receptor, *PR* progesterone receptor

^a^Assessed on 113 patients, according to St. Gallen consensus^11^

^b^Any setting

The median number of target lesions was 1 (range 1–3), with a median size of 21 mm (range 15–45 mm). The overwhelming majority of lesions—222 (92.9 %) of 239—were localized on the chest wall.

Forty-one patients (32.8 %) received chemotherapy in a neoadjuvant setting at the time of primary BC, while 62 patients (49.6 %) underwent chemotherapy in an adjuvant setting. Seventy-one patients (56.8 %) received adjuvant endocrine treatment. All patients had received at least one previous systemic treatment for metastatic disease. Specifically, 39 patients (31.2 %) received chemotherapy (median of two lines of treatment, range 1–6) and 69 patients received endocrine therapy (median two lines of treatment, range 1–3). Fifty-three patients (42.4 %) underwent adjuvant radiotherapy and 15 patients (12.0 %) were irradiated for the presence of skin metastases. As a result, 92 (38.5 %) of 239 target lesions in the present study were located in preirradiated skin. There were more previous systemic treatments in patients with triple negative (median 3, range 1–7) and *HER2* positive (median 3, range 2–6) BC than in patients with luminal A-like (median 1, range 0–6), luminal B-like (median 2, range 0–6), and luminal B-like, *HER2*-positive tumors (median 2, range 0–5, *P* = 0.042).

### Treatment

In 92 (73.6 %) of 125 patients, ECT was administered under general anesthesia or sedation, while local anesthesia was used in the remaining 33 patients (26.4 %). BLM was administered intravenously in 100 patients (80.0 %) and intratumorally in 25 (20.0 %). Of 239 tumors, 207 (86.6 %) were electroporated with a hexagonal-array needle electrode, 10 (4.2 %) with a linear-row needle electrode, 13 (5.4 %) with a plate electrode, and 9 (3.8 %) with multiple electrode types.

### Toxicity

No serious adverse events were reported during the procedure.

Toxicity data reported within the first 2 months are presented in Table 2. Paracetamol and nonsteroidal anti-inflammatory agents were effective in controlling postprocedural pain in all but four patients, who required narcotics, although as a single administration. The incidence of skin ulceration did not differ significantly depending on previous radiation (41.3 % of previous skin radiations vs. 30 % no previous skin radiation, *P* = 0.436). After the first ECT, 96 patients were asked if they would agree to receive another course of treatment, if required, and 96.9 % declared that they were potentially favorable.

**Table 2:** Toxicity within 2 months after electrochemotherapy (*n* = 125)

Toxicity	Any grade, *n* (%)	Grade 1, *n* (%)	Grade 2, *n* (%)	Grade 3, *n* (%)
Skin pain	79 (63.2)	28 (22.4)	38 (30.4)	13 (10.4)
Skin ulceration	41 (32.8)	17 (13.6)	14 (11.2)	10 (8.0)
Skin hyperpigmentation	34 (27.2)	23 (18.4)	11 (8.8)	–
Body odor	10 (8)	4 (3.2)	6 (4.8)	–
Nausea	10 (8)	10 (8)	0 (0)	0 (0)
Skin infection	9 (7.2)	6 (4.8)	2 (1.6)	1 (0.8)
Flulike symptoms	8 (6.4)	8 (6.4)	0 (0)	0 (0)
Fever	7 (5.6)	7 (5.6)	0 (0)	0 (0)
Rash	5 (4)	1 (0.8)	4 (3.2)	0 (0)
Soft tissue infection	2 (1.6)	0 (0)	2 (1.6)	0 (0)
Vomiting	2 (1.6)	2 (1.6)	0 (0)	0 (0)
Localized edema	1 (0.8)	0 (0)	1 (0.8)	0 (0)
Postoperative hemorrhage	1 (0.8)	0 (0)	1 (0.8)	0 (0)

### Tumor Response

**Fig. 1:**
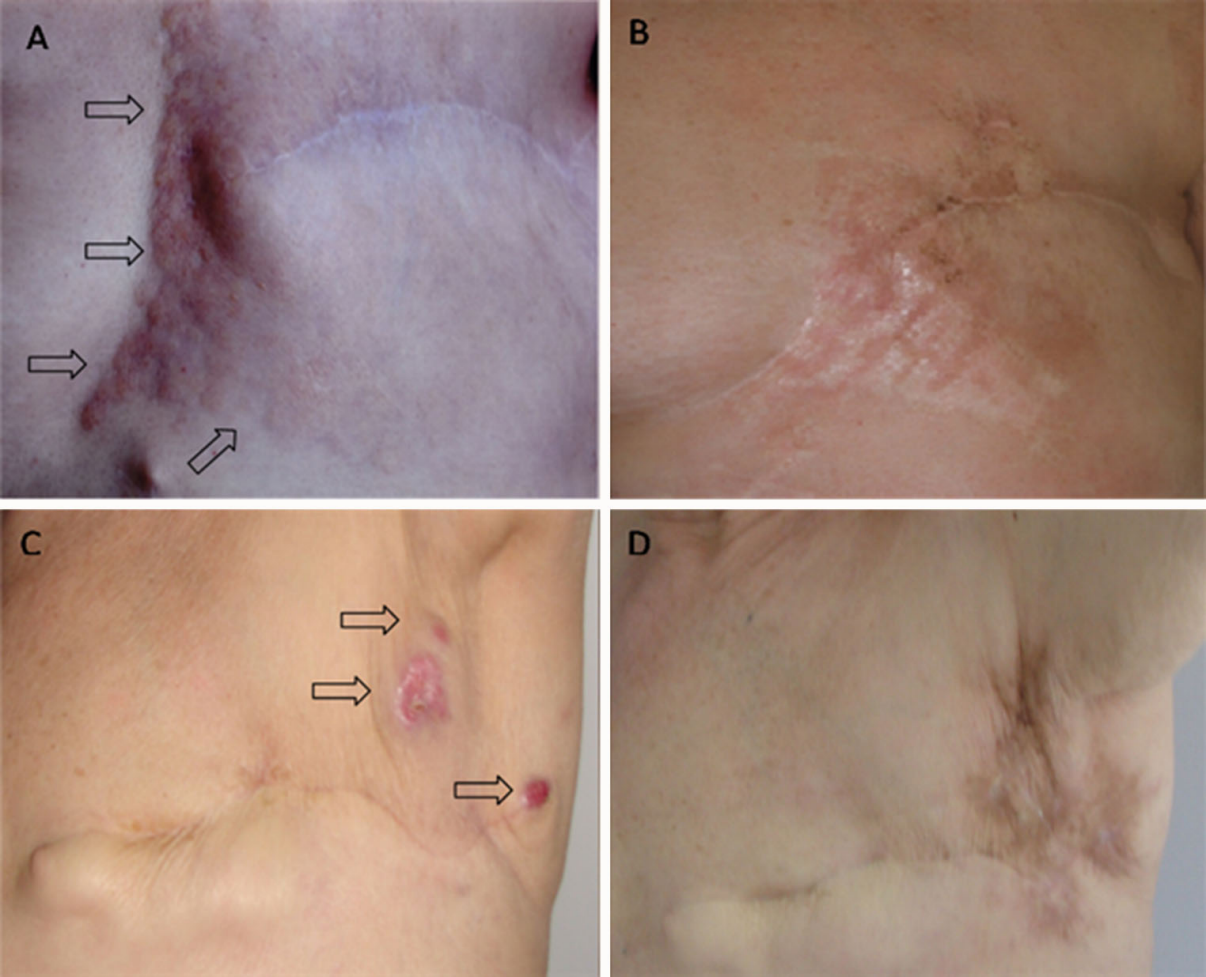
Skin metastases from BC treated with ECT in two patients. Baseline presentation (**a**, **c**) and 1-year follow-up (**b**, **d**). *Arrows* contour tumor spread or indicate skin metastases

Among 125 patients, the follow-up of 12 (9.6 %) was less than 2 months after ECT; these subjects were not evaluated for response. Therefore, 113 (90.4 %) of 125 patients and 214 (89.5 %) of 239 target lesions were evaluated for tumor response. Two months after ECT, per-tumor response was as follows: CR 68.5 %, partial response (PR) 23.5 %, stable disease 6.6 %, progressive disease 0.9 %, and not evaluable 0.5 % as a result of inflammatory reaction and crust formation. Accordingly, the OR rate was 92 % (Fig. 1).

Sixty-six patients (58.4 %) experienced CR, 36 (31.8 %) PR, 8 (7.1 %) stable disease, and 2 (1.8 %) had progressive disease; in 1 patient (0.9 %), tumor response was not evaluable as a result of local skin conditions. Overall, the per-patient OR rate was 90.2 %.

The variables associated with response are shown in Table 3. The CR rate was higher in small (<3 cm) rather than large (≥3 cm) tumors (80.3 vs. 46.1 %, *P* < 0.0001) and in patients without visceral metastases rather than in those with visceral involvement (80.5 vs. 55.0 %, *P* < 0.001). The CR rate was also higher among ER-positive (77.2 vs. 59.8 % in ER-negative, *P* = 0.006) and low proliferating tumors (Ki-67 < 14 %, 79.5 % vs. Ki-67 > 14 %, 58.8 %; *P* < 0.001). In multivariate analysis, tumor size <3 cm was confirmed to be the most powerful predictor of CR (*P* < 0.001), followed by the absence of visceral metastases (*P* = 0.001), ER-positive status (*P* = 0.016), and low Ki-67 (*P* = 0.024).

**Table 3:** OR for CR after electrochemotherapy

Variable	CR (%)	Univariate analysis			Multivariate analysis			1-year LPFS %	95 % CI	Multivariate analysis		
		OR	95 % CI	*P*	OR	95 % CI	*P*			HR	95 % CI	*P*
Size												
<3 cm (*n* = 55)	80.3	1.966	1.432–2.699	<0.001	7.22	3.35–15.57	<0.001	97.4	92.6–100	5.88	1.59–21.67	0.008
≥3 cm (*n* = 58)	46.1							75.6	63.9–89.4			
Ulceration												
No (*n* = 72)	73.7	1.285	1.035–1.595	0.008	1.59	0.74–3.41	0.234	85.6	76.5–95.8	1.21	0.44–3.30	0.712
Yes (*n* = 41)	55.7							87.3	77.3–98.6			
Receptor status												
ER positive (*n* = 65)	77.2	1.590	1.149–2.199	0.006	2.45	1.19–5.07	0.016	84.8	75.4–95.3	0.85	0.37–3.33	0.852
ER negative (*n* = 48)	59.8							88.8	80.1–98.6			
Ki-67												
<14 % (*n* = 58)	79.5	1.641	1.175–2.293	<0.001	2.38	1.12–5.07	0.024	90.2	82.2–98.9	0.85	0.41–3.29	0.786
>14 % (*n* = 55)	58.0							80.7	69.0–94.5			
Visceral metastases												
No (*n* = 67)	80.5	1.783	1.364–2.330	<0.001	3.60	1.66–7.79	0.001	86.5	77.4–96.7	1.98	0.72–5.50	0.185
Yes (*n* = 46)	55.0							86.0	76.0–97.3			

*CR* complete response, *OR* odds ratio, *CI* confidence interval, *LPFS* local progression-free survival, *HR* hazard ratio, *ER* estrogen receptor

The distribution of tumor response according to the BC intrinsic subtypes is presented in Table 4. The CR rate in patients with luminal A-like disease was significantly higher compared to all other subgroups (73.9 vs. 54.7 %, *P* = 0.02). There was no significant difference in tumor size among BC subtypes (*P* = 0.262).

**Table 4:** Tumor response to electrochemotherapy according to surrogate definition of breast cancer intrinsic subtypes

Response	Luminal A-like (*n* = 23), *n* (%)	Luminal B-like (*HER2* negative) (*n* = 22), *n* (%)	Luminal B-like (*HER2* positive) (*n* = 18), *n* (%)	Triple negative (*n* = 35), *n* (%)	*HER2* positive (*n* = 11), *n* (%)
CR	17 (73.9)	11 (50.0)	10 (55.6)	20 (57.1)	6 (54.5)
PR	4 (17.4)	9 (40.9)	5 (27.8)	11 (31.4)	5 (45.5)
SD	1 (4.3)	2 (9.1)	1 (5.6)	4 (11.4)	0 (0)
PD	1 (4.3)	0 (0)	1 (5.6)	0 (0)	0 (0)
NA	0 (0)	0 (0)	1 (5.6)	0 (0)	0 (0)

According to Goldhirsh et al.^11^; *n* = 109 (in four patients, there was no reliable pathologic information). Luminal A-like tumors (ER and PgR positive, *HER2* negative, Ki-67 low); luminal B-like, *HER2*-negative tumors (ER positive, *HER2* negative, Ki-67 high and/or PgR low or negative); luminal B-like, *HER2* positive tumors (ER positive, *HER2* overexpressed or amplified); *HER2* positive, nonluminal tumors (*HER2* overexpressed or amplified, ER and PgR negative); triple negative tumors (ER, PgR, and *HER2* negative)

*CR* complete response, *PR* partial response, *SD* stable disease, *PD* progressive disease, *NA* not assessable, *ER* estrogen receptor, *PR* progesterone receptor

There was no significant association between response and several clinical (patient age, *P* = 1.00; type of surgery on primary BC, *P* = 0.070; time from primary BC to recurrence, *P* = 0.269; presence of lymphedema, *P* = 0.636; previous radiation, *P* = 1.00) and procedural (anesthesiology technique, *P* = 0.377; electrode type, *P* = 0.799; route of BLM administration, *P* = 0.606; number of electric pulses, *P* = 0.842) parameters.

### Local Tumor Control

**Fig. 2:**
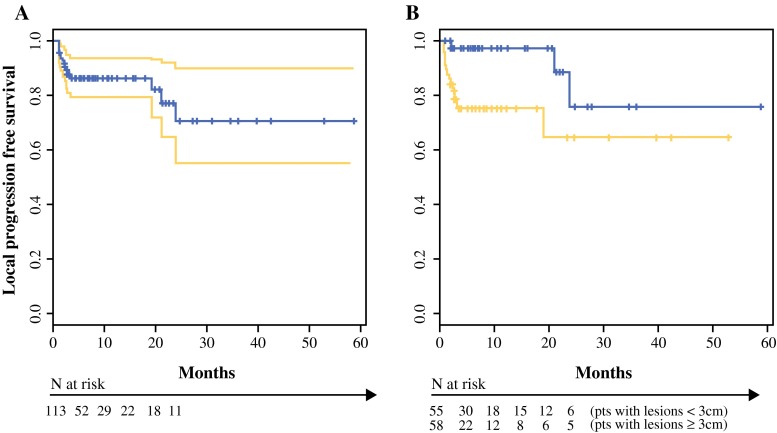
Tumor control after ECT. Kaplan–Meier curves for LPFS in **a** whole cohort and **b** subgroups of patients with lesions <3 cm (*blue line*) and ≥ 3 cm (*yellow line*)

Median follow-up time was 5.9 months (range 3–58 months). Median LPFS was not reached. One-year LPFS was 86.2 % (95 % confidence interval [CI] 79.3–93.8) (Fig. 2).

One-year LPFS in patients who experienced a CR was 96.4 % (95 % CI 91.6–100). In multiple Cox regression analysis, tumor size was the only significant prognostic factor for LPFS (Table 3).

One-year LPFS survival in patients with small (<3 cm) tumors was 97.4 % (95 % CI 92.6–100), whereas in those with larger tumors (≥3 cm), it was 75.6 % (95 % CI 63.9–83.4 *P* = 0.005).

## Discussion

This study showed for the first time that a subgroup of BC patients, identified by routinely used immunohistochemical markers, was particularly sensitive to ECT with BLM. To our knowledge, this cohort analysis was based on the largest series of BC patients treated by ECT to date.

ECT was mainly administered under general sedation or general anesthesia (74 % of patients), while the preferential route for BLM administration was intravenous infusion (80 % of patients). In most cases (89 %), treated tumors were managed using a hexagonal-array, 20 mm long needle electrode. The most frequently reported adverse effects were transient pain and dermatologic toxicity.^16^–^22^ Generally, treated skin develops a transient inflammatory reaction. Occasionally, erosions or ulcerations may occur, followed by crust formation. In case of tumor regression, the skin may appear slightly less pigmented, while in some patients it is possible to observe local skin hyperpigmentation, which is a well-known effect of BLM. In patients with locally advanced disease, tumor shrinkage after ECT may cause tissue ulceration requiring specialist wound care.^22^ Nevertheless, previous experience has demonstrated that effective management of cutaneous metastases provided symptomatic relief and better quality of life to patients.^20^

With the present study, we confirm the absence of systemic adverse effects of ECT, as well as a favorable toxicity profile (grade 3 ulceration in 8 % of patients, according to the meta-analysis of Spratt et al., grade 2 hyperpigmentation in 8.8 %), and a high level of acceptance.^7^ Patients experienced minimal discomfort and needed small amounts of postprocedural analgesics; further, only 10 % of adverse effects were severe, with the exception of transient pain within the first 48 h. As a result, 97 % of the 96 patients who were asked if they would agree to receive further treatment responded favorably. Our results are in line with the ESOPE study, where more than 90 % of patients declared that they were potentially amenable to treatment.^6^

Melanoma and BC represented more than 95 % of tumors included in two recently published meta-analyses where the indicated CR rates after ECT were 59 and 57.5 %, respectively.^7^,^23^ In the present study, the OR rate was 90.2 %, with a CR rate of 58.4 %, in agreement with the ESOPE study which reported an OR rate of 90.4 %, with 64.3 % of patients experiencing CR.^6^ A recently published clinical trial on 55 patients, representing the largest published retrospective experience with ECT in BC, showed a CR response rate of 40 % as the most favorable outcome among elderly patients.^22^ Consistent with a recent meta-analysis, in the present study the response to treatment in small tumors (<3 cm) was higher, similar to that seen in ER-positive, low-proliferating tumors (representing the luminal A-like BC subtype) and in patients without ulcerated lesions or visceral metastases.^24^

In particular, the CR rate in the luminal A-like BC subtype was 73.9 %, which was significantly higher than in triple-negative and *HER2* positive BC patients (57.1 and 54.5 %, respectively), independent of tumor size. However, although ECT in triple-negative BC in our series was used after failure of several lines of treatment and in conditioning a highly refractory disease, the CR rate in this group nonetheless exceeded 50 %.

We are aware that clinical evaluation may be a subjective assessment of tumor response. A pilot study including 11 patients with chest wall recurrence from BC investigated the role of ^18^F-fludeoxyglucose positron emission tomography (FDG-PET). This study indicates that not only FDG-PET/computed tomography (CT) but also dual time point imaging FDG-PET/CT is promising for evaluation and planning of ECT and could be useful for other localized anticancer treatments as well.^25^ On the other hand, this imaging technique, which is not widely available and which has nonnegligible costs, has a low sensitivity for small tumor deposits, limiting its application in cutaneous oncology.^26^

In our patients, data on local control indicated a 1-year LPFS of 86.2 % within the ECT field (Fig. 2), increasing to 96.4 % in those with CR. In our experience, small (<3 cm) tumor size represented the main predictor of local control compared to large (≥3 cm) tumor size (97.4 vs. 75.6 % at 1 year, respectively).

Skin involvement represents a less frequent but not uncommon event in the metastatic pattern of BC, accounting for 5–30 % of advanced cases in different series.^1^,^2^ In addition to their association with unfavorable prognosis, skin metastases cause strong psychologic distress.^16^ Surgical resection with a radical intent can only be offered to a limited number of patients as a result of multifocality and clinically occult lymphangitic spread.^27^ In these cases, radiotherapy is generally the best option, but it is often unfeasible on previously irradiated tissues and on lesions that have spread on a wide area. Lack of capillary distribution of radiologic facilities in the territory and the long duration of the entire cycle on multiple sessions may represent further criticisms. Conversely, ECT is applicable on preirradiated areas with the possibility to treat many lesions in a single session, without systemic side effects and a favorable toxicity profile. At any rate, ECT is repeatable and can even be performed in an outpatient setting.

Undoubtedly, our findings need broader and prospective confirmation. Furthermore, it will be necessary to clarify whether delaying progression of cutaneous metastases by ECT may provide clinically meaningful benefit to patients, such as delay of disease-related symptoms or preservation of quality of life. In general, the value of progression-free survival, as a surrogate marker for patient benefit, has recently been subjected to critical reappraisal.^28^ In fact, patient-centered outcomes will a crucial issue in future studies on ECT.^29^,^30^
